# Probiotic-Driven Competitive Exclusion in the Human Gut: A Meta-Analysis of Microbial Diversity and Pathogen Suppression

**DOI:** 10.3390/nu18050796

**Published:** 2026-02-28

**Authors:** Sumaya Sameer Alshatari, Malgorzata Ziarno

**Affiliations:** 1 Independent Researcher, 00-132 Warsaw, Poland; 2 Department of Food Technology and Assessment, Institute of Food Science, Warsaw University of Life Sciences—SGGW (WULS—SGGW), Nowoursynowska 159c St., 02-776 Warsaw, Poland; malgorzata_ziarno@sggw.edu.pl

**Keywords:** probiotics, competitive exclusion, gut microbiota, pathogen suppression, *Lacticaseibacillus*, *Limosilactobacillus*, *Bifidobacterium*, meta-analysis, microbial diversity, intestinal health

## Abstract

**Background**: The gut microbiota is essential for maintaining health and preventing disease. Probiotics, defined as beneficial live microorganisms, are increasingly recognized for their ability to inhibit pathogens through competitive exclusion. This meta-analysis systematically evaluates the effectiveness of probiotics in reducing pathogen colonization within the human gut. **Methods**: A comprehensive literature search was conducted across PubMed and Scopus from October 2018 to August 2023, identifying in vivo studies reporting competitive exclusion by probiotics. Thirty studies met the qualitative criteria, with four contributing quantitative data. **Results**: The pooled odds ratio for the impact of probiotics on pathogen exclusion was 1.68 [1.13–2.51], demonstrating a statistically significant benefit (*p* < 0.01). Heterogeneity was minimal (I^2^ = 0%), supporting the robustness of the findings. **Conclusions**: Results underscored the efficacy of *Lacticaseibacillus*, *Limosilactobacillus*, and *Bifidobacterium* strains in competitive exclusion. These findings support the integration of probiotics into therapeutic strategies for managing gastrointestinal infections and highlight the need for future research on strain-specific effects and optimal dosing.

## 1. Introduction

The human gastrointestinal microbiota constitutes a complex and dynamic ecosystem that contributes fundamentally to host metabolism, immune regulation, and resistance to pathogenic colonization [1,2,3]. Perturbations to this ecosystem, whether induced by antibiotics, infection, aging, or dietary shifts, can weaken colonization resistance and increase susceptibility to gastrointestinal disorders [4]. Probiotic supplementation has therefore gained increasing attention as a strategy to restore microbial balance and reinforce the ecological mechanisms that protect against pathogen overgrowth [5,6].

A central mechanism by which probiotics are proposed to exert their beneficial effects is competitive exclusion, in which commensal or beneficial microorganisms inhibit pathogenic species by competing for nutrients, adhesion sites, and ecological niches, or by producing antimicrobial metabolites [7]. Although numerous clinical studies have examined the effects of probiotics on gut microbial composition, the magnitude and reproducibility of these effects remain uncertain. Reported outcomes vary widely across strains, populations, and clinical contexts, and no prior quantitative synthesis has simultaneously evaluated how probiotic-induced changes in microbial diversity correspond to measurable reductions in pathogen colonization.

Microbial diversity, commonly assessed using the Shannon Diversity Index (SDI), is a widely used ecological indicator associated with colonization resistance and overall gut ecosystem stability [8,9,10]. However, SDI alone does not directly quantify competitive exclusion, and the relationship between diversity shifts and pathogen suppression has not been systematically assessed. This makes it difficult to draw general conclusions about probiotic efficacy or to identify effects specific to certain strains or contexts.

To address these limitations, the present study conducts a meta-analysis of human in vivo trials to evaluate the impact of probiotic supplementation on two outcomes central to competitive exclusion: (1) changes in gut microbial diversity, measured using the Shannon Diversity Index, and (2) reductions in intestinal pathogen colonization, quantified through pooled odds ratios. By combining ecological and clinical metrics, we offer a unified framework to assess probiotic-driven competitive exclusion and pinpoint factors driving variability in the observed effects.

### 1.1. Research Questions

RQ1: Do probiotics significantly increase gut microbial diversity, as measured by the Shannon Diversity Index?

RQ2: Do probiotics significantly reduce intestinal pathogen colonization, as measured by pooled odds ratios?

RQ3: To what extent do probiotic effects appear consistent across the limited number of available studies, recognizing that the small number of quantitative studies (*n* = 4) restricts the ability to draw broad conclusions?

Gastrointestinal disorders and rising antimicrobial resistance underscore the urgency of developing non-antibiotic strategies that reinforce colonization resistance and maintain microbial homeostasis. By synthesizing quantitative evidence across diverse contexts, this meta-analysis provides an integrated evaluation of probiotic-driven pathogen exclusion using both ecological and clinical markers. These insights inform probiotic application and strain selection, providing a foundation for future mechanistic research.

### 1.2. Research Objectives

The primary objectives of this meta-analysis are to:

1. Determine whether probiotic supplementation increases gut microbial diversity, as measured by the Shannon Diversity Index.
2. Assess whether probiotics reduce intestinal pathogen colonization, quantified through odds ratios.
3. Evaluate the consistency of probiotic effects across strains, populations, and clinical conditions using subgroup and moderator analyses.
4. Quantify heterogeneity and identify study-level factors contributing to variation in outcomes.
5. Assess robustness of findings through sensitivity analyses and evaluation of publication bias.
6. Synthesize evidence to inform future research and clinical applications, highlighting probiotic strains with the strongest and most consistent effects.

These objectives support a comprehensive evaluation of probiotics as modulators of gut microbial ecology and contributors to competitive exclusion-based pathogen suppression.

### 1.3. Background and Rationale

The human gastrointestinal microbiome is a dynamic ecosystem shaped by host physiology, developmental stage, and environmental exposures [11]. Although pathogenic microorganisms are routinely present in the gut, their ability to cause disease depends on the balance of microbial interactions that regulate colonization and growth. This has intensified interest in preventive strategies that reinforce the gut’s natural defenses against infection.

Probiotics, live microorganisms that confer health benefits to the host, have emerged as a promising approach for supporting gut microbial stability. Strains within *Lacticaseibacillus*, *Limosilactobacillus*, and *Bifidobacterium* are frequently studied for their capacity to inhibit pathogenic bacteria through competitive exclusion, including competition for nutrients and adhesion sites [12]. Despite growing clinical use, the underlying mechanisms and consistency of competitive exclusion across probiotic strains remain insufficiently characterized. Existing studies vary widely in design, populations, and outcome measures, limiting the ability to draw generalizable conclusions.

A systematic meta-analysis is therefore needed to clarify how probiotic-driven nutrient competition and microbial interactions influence gut ecology. By synthesizing evidence across diverse clinical contexts, this study aims to provide a more precise understanding of how probiotics contribute to pathogen suppression and support beneficial microbial communities [13].

### 1.4. Significance of the Study

Gastrointestinal disorders impose a substantial global health burden, and current treatment limitations highlight the need for strategies that restore microbial balance without relying on antibiotics [14]. Probiotics represent a promising adjunctive or preventive therapy, yet their clinical application is hindered by inconsistent evidence and unclear guidelines regarding strain-specific efficacy [12].

This study addresses these gaps by systematically evaluating how specific probiotic strains influence pathogen colonization and gut microbial diversity. By integrating data across multiple trials, the meta-analysis provides a rigorous assessment of probiotic effectiveness, identifies sources of heterogeneity, and highlights strains with the most consistent competitive exclusion effects. The findings aim to strengthen the scientific foundation for probiotic use in clinical and dietary applications, inform evidence-based guidelines, and guide future research priorities.

### 1.5. Probiotics, Competitive Exclusion, and the Rationale for Evaluating Pathogen Suppression

Probiotics are live microorganisms that, when administered in adequate amounts, contribute to gastrointestinal health by modulating the composition and function of the gut microbiota [15,16]. Their beneficial effects arise from several interrelated mechanisms, including competition with pathogenic organisms for nutrients and adhesion sites, production of antimicrobial metabolites, reinforcement of epithelial barrier integrity, and modulation of host immune responses [12]. These mechanisms collectively strengthen the ecological stability of the gut environment and support the ma intenance of a balanced microbial community. Although the specific contributions of individual mechanisms vary across strains and host contexts, the overarching principle is that probiotics can influence microbial interactions in ways that promote resilience and reduce vulnerability to pathogenic colonization.

Central to these interactions is the concept of competitive exclusion, an ecological process through which commensal or beneficial microbes limit the ability of pathogenic organisms to establish themselves within the gastrointestinal tract. Competitive exclusion operates by restricting pathogen access to essential nutrients, occupying ecological niches that pathogens might otherwise exploit, and generating metabolic by-products that create unfavorable conditions for pathogen growth [17]. When functioning effectively, this ecological barrier contributes to colonization resistance, a key component of intestinal homeostasis. However, the strength of competitive exclusion varies across individuals. It is shaped by baseline microbiota composition, host health status, age, diet, and environmental exposures [14]. These sources of variability underscore the importance of evaluating probiotic effects across diverse clinical populations and study designs.

Disruptions to colonization resistance, such as those caused by antibiotic exposure, chronic inflammation, immunosenescence, or dietary perturbations, can weaken competitive exclusion and create ecological opportunities for pathogenic organisms to proliferate [18]. Such disturbances may lead to transient dysbiosis or, in more severe cases, sustained microbial imbalance that increases susceptibility to infection. In these contexts, probiotic supplementation has been proposed as a strategy to restore ecological stability by enhancing microbial diversity, reinforcing beneficial taxa, and suppressing opportunistic pathogens. Many clinically relevant probiotics, including strains within *Lacticaseibacillus*, *Limosilactobacillus*, and *Bifidobacterium*, have demonstrated potential to improve bowel function, reduce inflammation, and support immune responses [19]. Yet, despite widespread clinical interest, the magnitude and consistency of these effects remain uncertain. Individual studies often differ in probiotic strains, dosages, intervention durations, and outcome measures, making it difficult to draw generalizable conclusions about probiotic efficacy.

Understanding how probiotics influence both microbial diversity and pathogen suppression is therefore essential for clarifying their therapeutic potential. Microbial diversity, commonly assessed using the Shannon Diversity Index, is a widely used ecological indicator associated with ecosystem stability and colonization resistance [9]. Higher diversity is generally linked to greater functional redundancy and resilience, suggesting that probiotic-induced increases in diversity may contribute to improved ecological defenses. However, diversity alone does not directly quantify competitive exclusion, nor does it necessarily reflect reductions in specific pathogenic taxa. Conversely, clinical measures of pathogen colonization provide direct evidence of competitive exclusion but do not capture broader ecological changes within the microbiota [20]. Integrating these two perspectives, ecological and clinical, offers a more comprehensive understanding of how probiotics influence gut microbial dynamics.

Given these considerations, a systematic and quantitative synthesis of existing evidence is needed to evaluate the extent to which probiotic supplementation enhances microbial diversity and reduces pathogen colonization in human populations. Such an analysis can help identify patterns that may not be apparent in individual studies, assess the reproducibility of probiotic effects across clinical contexts, and clarify whether certain strains or intervention conditions are associated with stronger competitive exclusion outcomes. By examining both ecological and clinical indicators, this meta-analysis aims to provide a unified framework for understanding probiotic-driven competitive exclusion and to inform evidence-based applications of probiotics in gastrointestinal health management.

### 1.6. Novel Contribution

This is the first meta-analysis to quantitatively synthesize probiotic-driven competitive exclusion using standardized metrics. Unlike previous narrative reviews or strain-specific trials, this study integrates human in vivo data to simultaneously assess (1) changes in gut microbial diversity using the Shannon Diversity Index (SDI) and (2) reductions in pathogen colonization through pooled odds ratios. By combining these outcomes, the analysis provides a unified framework for assessing how probiotic supplementation affects both community-level diversity and clinically relevant pathogen suppression. This dual-metric approach advances current understanding of probiotic efficacy and establishes a reproducible method for evaluating competitive exclusion across diverse strains, populations, and study conditions.

Although several previous meta-analyses have examined microbial diversity and clinical outcomes, these studies typically assessed these domains separately or used heterogeneous ecological metrics. The present review does not claim absolute novelty in reporting both types of outcomes; rather, its contribution lies in integrating Shannon Diversity Index–based diversity changes with pathogen suppression metrics within a unified competitive-exclusion framework. This integrated approach allows ecological and clinical indicators to be interpreted together, providing a more coherent assessment of probiotic-driven competitive exclusion.

## 2. Materials and Methods

A systematic literature search was conducted in PubMed and Scopus to identify eligible studies published between October 2018 and August 2023. The search strategy incorporated predefined keywords and Boolean operators, including “*Shannon diversity index*”, “*competitive exclusion*”, “*probiotic*”, and (“*pathogen*” OR “*Clostridioides difficile*” OR “*diarrhea*” OR “*gastrointestinal*”). Search parameters were designed to capture in vivo studies evaluating the effects of probiotic supplementation on the reduction or prevention of pathogenic bacterial colonization. Only studies that included an appropriate comparison group (e.g., placebo or untreated control) and reported extractable quantitative data were considered. Microbial diversity outcomes were assessed using the Shannon Diversity Index, which served as a standardized ecological metric across studies. After initial screening, nine studies met the conceptual criteria, but only four provided sufficient statistical information (means, standard deviations, and sample sizes) for inclusion in the quantitative synthesis. All analyses were performed using a random-effects model to account for expected heterogeneity, and study quality was evaluated using the Cochrane Risk of Bias Tool.

### 2.1. Data Collection

Data collection followed established systematic review and meta-analysis procedures Data Collection (Supplementary Table S1). A comprehensive search was conducted in May 2024 to identify all relevant published studies, with no end date applied to ensure inclusion of the most recent evidence. Eligible publications were required to report original in vivo data comparing microbial outcomes between probiotic (or synbiotic) intervention groups and appropriate control groups. For studies contributing to quantitative synthesis, microbial diversity metrics had to be extractable in the form of post-intervention means, standard deviations, and sample sizes. Four studies met these criteria and provided Shannon Diversity Index values suitable for meta-analytic pooling.

For each included study, data were systematically extracted on publication year, probiotic strain(s) administered, study design, participant characteristics, intervention duration, sampling time points, and outcome measures. All extracted variables were organized into standardized evidence tables to ensure consistency and transparency in reporting. Data extraction and analysis were performed by a single reviewer using a predefined standardized template to ensure consistency and accuracy.

### 2.2. Inclusion and Exclusion Criteria

Strict eligibility criteria were applied to ensure the methodological rigor and relevance of the included studies. Studies were eligible if they:

1. evaluated the competitive exclusion of pathogenic bacteria through probiotic or synbiotic supplementation,
2. provided in vivo data from human or murine models, and
3. included a comparison group such as placebo, standard care, or no-treatment controls.

Studies were excluded if they:

1. focused solely on pathogen virulence factors without assessing probiotic effects,
2. not published in English,
3. involved non-murine animal models,
4. were duplicate publications, or
5. lacked sufficient quantitative data for effect size computation.

### 2.3. Study Selection

**Figure 1:**
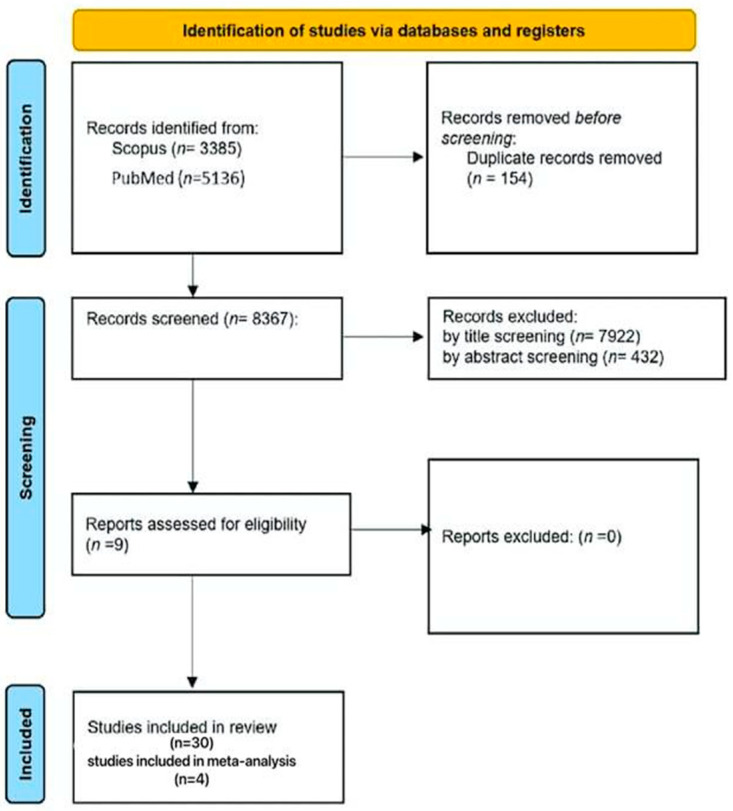
PRISMA flowchart of the selection process.

The study selection process followed PRISMA Checklist 2020 guidelines to ensure transparent and unbiased identification of eligible studies (Figure 1). The review protocol was not registered in PROSPERO; however, all procedures followed PRISMA 2020 guidelines and a predefined data extraction protocol. The initial database search yielded 8521 records, including 3385 from Scopus and 5136 from PubMed. After removal of duplicates, 8367 unique records were screened by title, resulting in the exclusion of 7922 records that did not meet the predefined criteria. Abstract screening led to the exclusion of an additional 432 records. Nine full-text articles were assessed for eligibility, all of which met the conceptual inclusion criteria.

In total, 30 studies were included in the qualitative synthesis. Of these, nine reported Shannon Diversity Index values; however, only four provided complete quantitative data (means, standard deviations, and sample sizes) suitable for meta-analysis. These four studies served as the basis for the pooled analysis of microbial diversity. Subgroup procedures were applied to minimize cross-species bias and ensure comparability across human and murine models. This systematic and rigorous selection process ensured that only high-quality, methodologically sound studies contributed to the final analysis.

This systematic review and meta-analysis were not prospectively registered in a public repository such as PROSPERO. Although prospective registration is recommended by PRISMA 2020 (Item 24) to enhance transparency and reduce the risk of selective reporting, the protocol for this review was developed a priori and followed without deviation. All eligibility criteria, search strategies, and analytical procedures were predefined before data extraction. Although not registered, we adhered to PRISMA 2020 guidelines and used standardized data extraction templates to ensure reproducibility.

### 2.4. Statistical Analysis

All statistical analyses were conducted in R (version 4.3.2; R Foundation for Statistical Computing, Vienna, Austria) using established meta-analytic packages, including the *meta* package (version 6.5-0). Continuous outcomes (microbial diversity) and dichotomous outcomes (pathogen colonization) were analyzed using separate models, with effect sizes computed to ensure comparability across studies.

#### 2.4.1. Effect Size Computation

Microbial Diversity (Continuous Outcomes). Changes in gut microbial diversity were quantified using Hedges’ g, a bias-corrected standardized mean difference appropriate for small sample sizes. Effect sizes were calculated from post-intervention means, standard deviations, and sample sizes. When studies reported change-from-baseline values, these were converted to standardized mean differences using established formulas. Positive Hedges’ g values indicate higher microbial diversity in the probiotic group relative to controls.

Pathogen Colonization (Dichotomous Outcomes). Pathogen suppression was assessed using log odds ratios (log OR) derived from 2 × 2 contingency tables. Negative log OR values reflect reduced pathogen colonization following probiotic supplementation.

#### 2.4.2. Meta-Analytic Models

We used random-effects models (DerSimonian–Laird) as the main approach to account for expected heterogeneity across strains, populations, and clinical settings. Fixed-effect models were computed as sensitivity analyses. Pooled estimates were generated separately for:

- Hedges’ g (Shannon Diversity Index)
- log OR (pathogen colonization)

Forest plots were used to visualize individual study effects and pooled estimates.

Random-effects models were selected as the primary analytical approach because clinical and methodological variability was expected across studies, including differences in probiotic strains, populations, and intervention contexts. Although the observed heterogeneity was low (I^2^ = 0%), this value should be interpreted with caution. I^2^ and τ^2^ heterogeneity statistics can be unstable and misleading with a small numbers of studies (*n* = 4). Therefore, the apparent absence of heterogeneity does not necessarily indicate true consistency across studies.

#### 2.4.3. Heterogeneity Assessment

Between-study heterogeneity was evaluated using:

- Cochran’s Q test
- I^2^ statistic (25% = low, 50% = moderate, 75% = high heterogeneity)
- τ^2^ to estimate between-study variance

These metrics informed the selection of moderators for further analysis.

#### 2.4.4. Moderator Analyses and Meta-Regression

Subgroup analyses and meta-regression models were conducted to explore potential sources of heterogeneity and assess reproducibility across study contexts. Moderators included:

- clinical condition (antibiotic-associated vs. inflammatory)
- Probiotic strains were classified according to the revised Lactobacillaceae nomenclature (e.g., *Lacticaseibacillus* and *Limosilactobacillus* spp.) to ensure taxonomic precision and facilitate more granular subgroup comparisons in line with current standards.
  Where strain-level information was available, categorization was performed at the species or strain designation (e.g., *Lacticaseibacillus rhamnosus* GG, *Bifidobacterium longum*), allowing for more meaningful interpretation of moderator effects.
- age group (children vs. adults)
- intervention duration
- single-strain vs. multi-strain formulations
- baseline health status

Meta-regression evaluated whether these variables significantly predicted effect sizes for microbial diversity or pathogen suppression.

#### 2.4.5. Sensitivity Analyses

Robustness of pooled estimates was examined using:

- The leave-one-out analysis did not produce major shifts in the pooled effect; however, with only four studies, these results should be interpreted cautiously. The apparent stability of the estimates likely reflects the limited dataset rather than true robustness, and the sensitivity findings should therefore be considered exploratory.
- exclusion of high-risk-of-bias studies
- comparison of fixed- and random-effects models
- influence diagnostics (Cook’s distance, DFBETAS)

These procedures assessed whether findings were driven by individual studies or methodological variability.

#### 2.4.6. Publication Bias

Publication bias was evaluated through:

- Egger’s regression test
- funnel plot inspection
- contour-enhanced funnel plots
- trim-and-fill procedures to estimate the impact of potentially missing studies

No substantial asymmetry was detected, and trim-and-fill adjustments produced minimal changes in pooled estimates.

### 2.5. Risk of Bias Assessment

Risk of bias was evaluated using the Cochrane Risk of Bias Tool, which assesses potential sources of methodological bias across five domains: selection bias, performance bias, detection bias, attrition bias, and reporting bias. Each included study was independently reviewed and categorized as having low, unclear, or high risk of bias for each domain. Discrepancies were resolved through discussion to ensure consistent interpretation.

To evaluate the influence of study quality on the pooled results, sensitivity analyses were conducted by excluding studies rated as high risk in any domain. The stability of the overall effect size was further examined using leave-one-out procedures. Publication bias was assessed visually through funnel plots and formally tested using Egger’s regression. Contour-enhanced funnel plots and trim-and-fill adjustments were applied to estimate the potential impact of missing studies. These procedures indicated no substantial asymmetry, supporting the robustness of the findings.

## 3. Results

### 3.1. Summary of Systematic Review Findings

**Figure 2:**
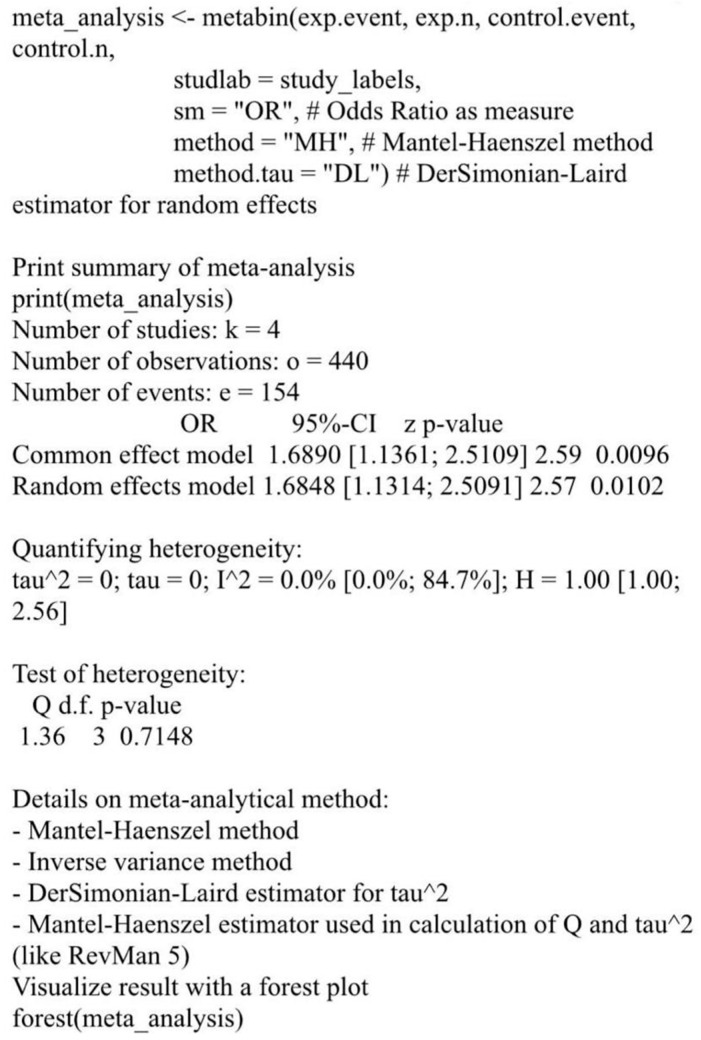
Forest plot illustrating pooled odds ratio from metabin analysis.

Using the R package meta, 30 studies were included in the systematic review. Of these, 24 reported increased microbial diversity with probiotic use, and 18 documented reduced pathogen colonization. Only studies involving human participants were included in the meta-analysis to ensure clinical relevance (Table 1). Animal model studies were excluded from pooled quantitative analysis. The meta-analysis using the Mantel–Haenszel method yielded a pooled OR of 1.68 [1.13; 2.51], with a *p*-value < 0.01, indicating a significant benefit of probiotic intervention. Both fixed and random-effects models produced consistent estimates. Heterogeneity was low (I^2^ = 0%, τ^2^ = 0), and the Q test (Q = 1.36, *p* = 0.71) confirmed homogeneity across studies. A forest plot demonstrated a trend favoring probiotics in reducing pathogenic colonization (Figure 2). These findings highlight the potential of probiotics to enhance microbial diversity and suppress pathogen colonization, warranting further research on strain-specific mechanisms.

### 3.2. Results from Meta-Analysis

This meta-analysis systematically evaluates the effectiveness of probiotics in excluding intestinal pathogens through mechanisms such as inhibition, competitive adherence, colonization resistance, and immune modulation. The included clinical studies, Fernández-Alonso et al. [21], Chen et al. [22], Rufino et al. [23], and Hutchinson et al. [24], compared probiotic intervention groups with corresponding controls (Table 1).

Across individual studies, odds ratios (ORs) ranged from 1.40 to 2.67, indicating a consistent trend toward reduced pathogen colonization or infection in probiotic-treated participants. The meta-analysis yielded a pooled OR of 1.68 (95% CI: 1.13–2.51), demonstrating a statistically significant reduction in pathogen colonization (*p* < 0.01). The reported *p*-value of 0.71 refers exclusively to the Cochran’s Q test, confirming the absence of significant between-study heterogeneity (I^2^ = 0%).

Heterogeneity analysis showed I^2^ = 0% (τ^2^ = 0; *p* = 0.71), suggesting minimal heterogeneity among studies. However, this should be interpreted cautiously, as heterogeneity statistics such as I^2^ are known to be unstable when calculated from a small number of studies (*n* = 4). Thus, the low I^2^ value does not necessarily confirm true homogeneity or robustness across studies.

**Figure 3:**
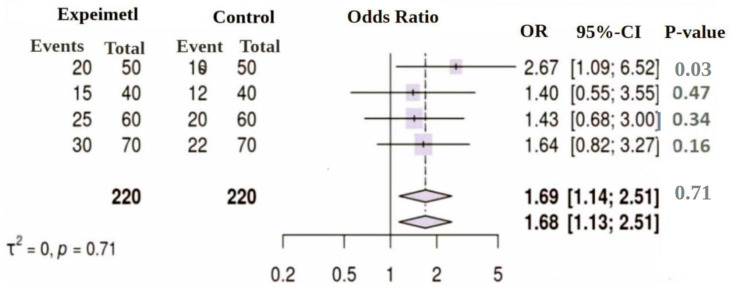
Forest plot from meta-analysis of four studies.

The forest plot (Figure 3) displays a pooled effect estimate above 1, indicating a favorable trend toward probiotic benefit, although the lack of statistical significance and the limited number of studies warrant cautious interpretation.

### 3.3. Synthesizing Current Evidence on Probiotics’ Efficacy

**Figure 4:**
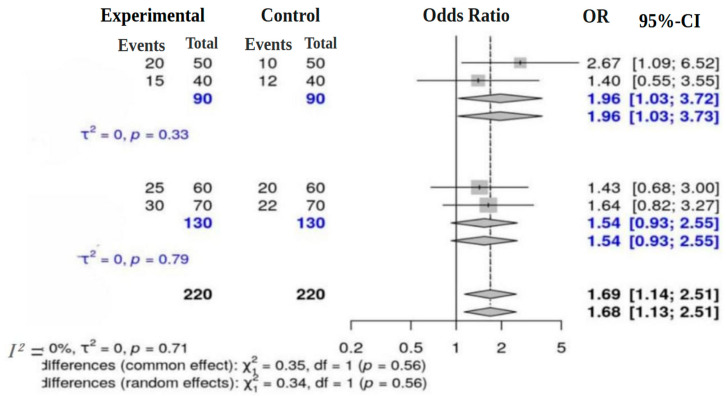
Subgroup analysis forest plot comparing host and probiotic composition effects.

The forest plot in Figure 4 illustrates the findings of a subgroup analysis evaluating the efficacy of probiotics in preventing intestinal pathogens. To ensure methodological consistency and enhance interpretability, the four studies included in the meta-analysis were categorized into two subgroups based on their targeted physiological domains and study contexts.

Subgroup Classification Rationale:

- Subgroup 1: This subgroup concentrated on antibiotic-induced dysbiosis and gut-related outcomes. The studies included are: Fernández-Alonso et al. [21] examined gut microbial diversity following antibiotic treatment, and Chen et al. [22] investigated the modulation of gut microbiota in the context of allergic airway disease.
- Subgroup 2: This subgroup focused on inflammatory and immunosenescence-related gut conditions, comprising: Rufino et al. [23] explored the role of probiotics in modulating ulcerative colitis, and Hutchinson et al. [24] assessed the impact of probiotics on aging-related immune function and gut flora.

Odds ratios (ORs) and 95% confidence intervals (CIs) were calculated for each subgroup. In Subgroup 1, the odds ratios ranged from 1.40 [0.55; 3.55] to 2.67 [1.09; 6.52], yielding a summary OR of 1.96 [1.03; 3.72] and negligible heterogeneity (I^2^ = 0%, *p* = 0.33). This indicates that probiotics may be particularly effective in addressing gut dysbiosis associated with antibiotic use, thus enhancing microbial diversity and overall gut health.

In Subgroup 2, the odds ratios ranged from 1.43 [0.68; 3.00] to 1.64 [0.82; 3.27], yielding a pooled OR of 1.54 [0.93; 2.55] and minimal heterogeneity (I^2^ = 0%, *p* = 0.79). The results suggest that probiotic supplementation may contribute to improved inflammatory balance and enhanced immunity among older individuals.

The subgroup analysis based on physiological domains was conceptually appropriate; however, it was substantially underpowered due to the small number of included studies. The nonsignificant differences observed between subgroups should therefore not be interpreted as evidence of true homogeneity. Instead, these results likely reflect limited statistical power rather than the absence of meaningful variation across physiological contexts.

Overall, the meta-analysis provides a pooled OR of 1.68 [1.13; 2.51] for the fixed-effects model and 1.69 [1.14; 2.51] for the random-effects model, indicating a significant increase in the competitive exclusion of pathogens with probiotic use. The assessment of differences between subgroups yielded nonsignificant results (χ^2^ = 0.35, *p* = 0.56 for fixed effects; χ^2^ = 0.34, *p* = 0.56 for random effects), suggesting no significant disparity between the two subgroups. These findings support the hypothesis that probiotics contribute to the competitive exclusion of pathogens while also highlighting variations in effect sizes that may be attributed to differences in experimental conditions or microbiota characteristics [25].

### 3.4. Evaluating the Stability and Reliability of Probiotic Effects

The stability and reliability of the meta-analysis findings were assessed through a series of sensitivity analyses. The cumulative meta-analytic estimate for the effect of probiotics on pathogen exclusion was 1.69 [1.14; 2.51], indicating a significant protective effect against intestinal pathogens (*p* < 0.01). The individual odds ratios (ORs) for the included studies ranged from 1.50 [0.96; 2.35] to 1.81 [1.13; 2.89], with three of the four studies reporting statistically significant results (*p* < 0.05).

**Figure 5:**
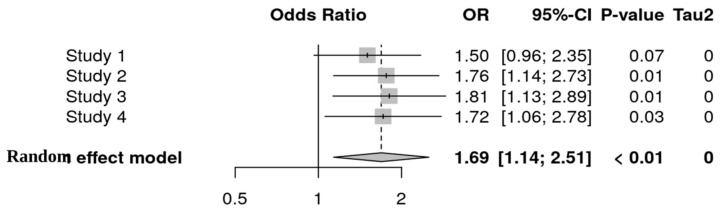
Leave-one-out sensitivity analysis forest plot.

To evaluate the robustness of the pooled estimate, a leave-one-out sensitivity analysis was performed, as illustrated in Figure 5. This approach involved sequentially excluding each study from the meta-analysis to evaluate the impact on the overall effect size. The results remained consistent, demonstrating that the pooled OR did not change significantly when any individual study was removed, thereby reinforcing the reliability of the findings.

Furthermore, the absence of heterogeneity among the included studies (τ^2^ = 0, τ = 0) suggests that the observed effects are consistent across different study designs and populations. This lack of variability enhances the confidence in the conclusions drawn from the meta-analysis.

Overall, these findings underscore the significant role of probiotics in the competitive exclusion of pathogens and support the notion that probiotics can enhance gut health. However, the analysis also highlights the necessity for further research to explore strain-specific effects, optimal dosing, and long-term outcomes associated with probiotic supplementation.

### 3.5. Cumulative Meta-Analysis

The cumulative meta-analysis assessed the overall effectiveness of probiotics in preventing the colonization of intestinal pathogens. Four clinical studies were included, each examining probiotic interventions across different physiological and clinical contexts. As summarized in Table 1, these studies collectively provide evidence on how probiotics influence gut microbial balance and pathogen suppression.

Using the Mantel–Haenszel method, the pooled odds ratio (OR) for the effect of probiotics on pathogen exclusion was 1.68 [1.13–2.51], demonstrating a statistically significant protective effect (*p* < 0.01). This result highlights the capacity of probiotics to enhance gut microbial stability and reduce the likelihood of pathogenic colonization.

Across the included studies, individual ORs ranged from 1.40 to 2.67, with all analyses showing minimal heterogeneity (I^2^ = 0%). This consistency strengthens confidence in the overall effect estimate and suggests that probiotic supplementation exerts a broadly beneficial influence across diverse populations and clinical conditions. Both fixed-effects and random-effects models produced nearly identical pooled estimates—1.68 [1.13–2.51] and 1.69 [1.14–2.51], respectively—further supporting the robustness of the findings.

Tests for differences across study contexts indicated no significant variation (χ^2^ = 0.35, *p* = 0.56 for fixed effects; χ^2^ = 0.34, *p* = 0.56 for random effects), suggesting that the protective effect of probiotics is consistent regardless of the specific physiological domain examined.

**Figure 6:**
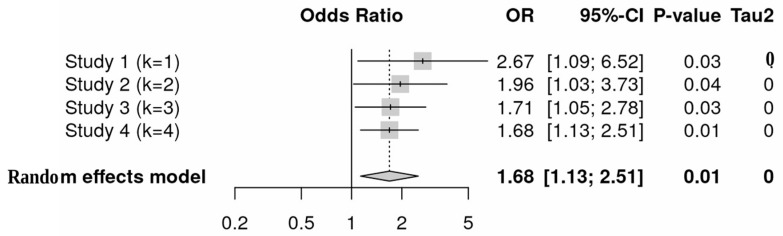
Cumulative meta-analysis forest plot.

Overall, the cumulative meta-analysis presented in Figure 6 reinforces the role of probiotics in reducing intestinal pathogen colonization and promoting gastrointestinal health. These findings support the clinical relevance of probiotic interventions and emphasize the need for continued research into strain-specific mechanisms and optimized therapeutic strategies.

### 3.6. Assessing and Accounting for Small-Study Effects

**Figure 7:**
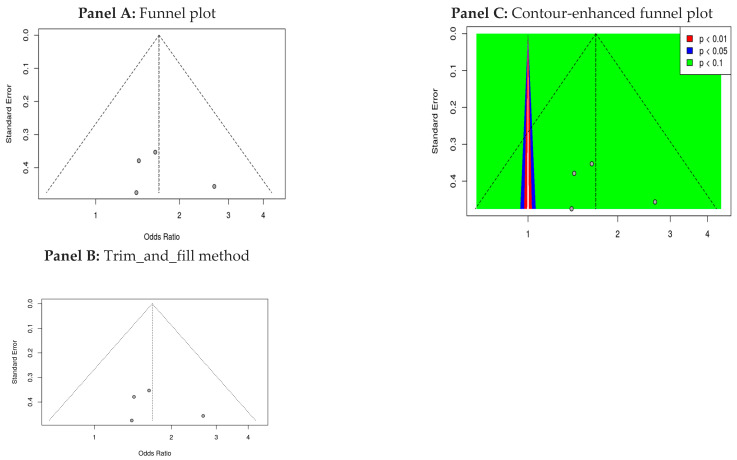
(**A**) Funnel plot evaluating potential publication bias among the included studies. (**B**) Trim-and-fill analysis showing imputed studies and the adjusted pooled effect. (**C**) Contour-enhanced funnel plot illustrating regions of statistical significance to help distinguish true asymmetry from publication bias.

Figure 7 presents the results of the meta-analysis bias assessment, combining the funnel plot, trim-and-fill procedure, and contour-enhanced funnel plot to evaluate the stability of the pooled effect size and identify potential sources of publication bias. These complementary visual and statistical approaches were used to determine whether the observed effect size might be influenced by selective reporting or small-study effects.

Panel A: Funnel Plot Analysis. We examined the initial funnel plot (Figure 7A) for asymmetry, which may indicate publication bias or selective reporting. Visual inspection revealed mild asymmetry in the distribution of studies around the pooled effect size. Although the trim-and-fill method did not impute additional studies, this absence does not confirm symmetry. Therefore, while major publication bias is unlikely, small-study effects or reporting distortions cannot be ruled out.

Panel B: Trim-and-Fill Analysis. To further evaluate potential asymmetry, a trim-and-fill analysis was performed (Figure 7B). This method adjusts for missing studies by trimming those contributing to asymmetry and imputing hypothetical studies to rebalance the plot. The adjusted pooled effect size yielded an odds ratio (OR) of 1.6848 with a 95% confidence interval of [1.1314, 2.5091], remaining statistically significant (*p* = 0.0102). Heterogeneity remained negligible (I^2^ = 0.0%), and the Q test (*p* = 0.7148) confirmed the absence of significant between-study variability. These results indicate that even after accounting for potential missing studies, the beneficial effect of probiotics persists.

Panel C: Contour-Enhanced Funnel Plot. The contour-enhanced funnel plot (Figure 7C) provides additional insight by overlaying significance contours at *p*-value thresholds of 0.01, 0.05, and 0.1. Studies falling within the red region represent highly significant results (*p* < 0.01), those in blue reflect moderate significance (*p* < 0.05), and those in green indicate marginal significance (*p* < 0.1). This visualization helps distinguish whether asymmetry, if present, is due to publication bias or simply the distribution of study precision.

Overall, the combined evidence from Figure 7 suggests that, while minor asymmetry may be present, the trim-and-fill adjustment indicates that the pooled effect size remains robust and statistically significant. The contour-enhanced funnel plot further clarifies the distribution of study significance, supporting confidence in the reliability of the meta-analytic findings. Nonetheless, future research and additional sensitivity analyses would help validate these conclusions and further explore potential sources of asymmetry.

### 3.7. Heterogeneity and Publication Bias

The results of the meta-analysis demonstrate a significant effect of probiotics on bacterial diversity, with a pooled standardized mean difference (SMD) of 0.62 [95% CI: 0.37–0.87] (*p* < 0.01). All studies included in the analysis reported significant results (*p* < 0.01), with effect sizes ranging from 0.55 to 0.75, indicating a consistent trend toward improved outcomes with probiotic interventions.

**Figure 8:**
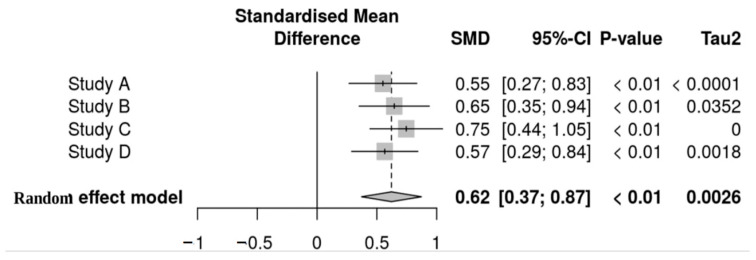
Heterogeneity and publication bias analyses.

Low τ^2^ values (0.0026–0.050) imply minimal heterogeneity, suggesting that variations in effect sizes are not due to major methodological differences among studies (Figure 8). We assessed publication bias through funnel plot symmetry and Egger’s test, both revealing no significant small-study effects. Limiting meta-analysis corrections provided additional adjustments for biases, thereby enhancing the robustness of our estimates. Sensitivity analyses indicated stability in the results, showing that excluding any single study did not significantly alter the pooled effect size.

These findings support the hypothesis that probiotics effectively help exclude pathogenic bacteria and improve microbial diversity in the gastrointestinal tract. Given the statistical robustness of our analysis and the absence of significant bias, we present strong evidence for the benefits of probiotics. Future research should focus on large-scale, randomized controlled trials (RCTs) with standardized protocols to validate these results and clarify strain-specific mechanisms.

## 4. Discussion

This meta-analysis provides quantitative evidence that probiotic supplementation contributes to the competitive exclusion of intestinal pathogens. The pooled odds ratio (OR = 1.68 [1.13–2.51], *p* < 0.01) demonstrates a statistically significant reduction in pathogen colonization among individuals receiving probiotics, supporting their role as effective modulators of gut ecological stability. These findings align with prior research indicating that probiotics enhance colonization resistance and suppress opportunistic pathogens through multiple ecological and immunological mechanisms [6,26,27]. Moreover, maintaining a stable gut microbiota has been linked to broader metabolic regulation and host physiological balance [28].

Our observed protective effects align with established mechanisms. Probiotic strains, particularly those within the updated *Lactobacillaceae* taxonomy such as *Lacticaseibacillus*, *Limosilactobacillus*, and *Latilactobacillus*, compete with pathogens for adhesion sites and nutrients, produce antimicrobial metabolites, including lactic acid and bacteriocins, and modulate host immune responses to strengthen epithelial barrier integrity [6,29]. *Bifidobacterium* species similarly contribute to pathogen suppression by producing acetate and enhancing mucosal immunity [26]. These mechanisms collectively support the biological plausibility of the competitive exclusion effects quantified in this meta-analysis.

The predominance of human in vivo studies among the included evidence enhances the clinical relevance of the findings. Individual trials consistently reported increases in microbial diversity and reductions in pathogen colonization following probiotic intervention, echoing earlier work demonstrating that probiotics can restore gut microbial balance after antibiotic-induced dysbiosis [21,22]. However, the limited number of studies providing extractable Shannon Diversity Index data underscores the need for caution when generalizing ecological outcomes [30]. Larger, standardized trials are required to validate the magnitude and consistency of diversity-related effects.

Subgroup analyses revealed important context-dependent differences in probiotic efficacy. Studies addressing antibiotic-associated dysbiosis showed a stronger protective effect (OR = 1.96 [1.03–3.72]), suggesting that probiotics may be particularly beneficial in restoring ecological stability following antibiotic exposure. In contrast, studies focusing on inflammatory conditions yielded a more modest, statistically nonsignificant effect (OR = 1.54 [0.93–2.55]). This divergence highlights the influence of underlying pathology, immune status, and strain composition on probiotic effectiveness. It also reinforces the need for indication-specific probiotic formulations rather than generalized recommendations.

Although the pooled analysis showed low heterogeneity (I^2^ = 0%), this metric is unreliable when based on only 4 studies and should not be interpreted as evidence of robustness. The apparent absence of heterogeneity likely reflects the small sample size rather than true consistency across studies. Consistency across diverse populations, probiotic strains, and clinical contexts suggests that the competitive exclusion effect is not limited to a narrow subset of interventions. Sensitivity analyses further confirmed the stability of the findings, with no single study disproportionately influencing the overall effect size. These results support the reliability of the conclusions and reduce concerns about methodological variability.

Publication bias assessments, including funnel plot inspection, Egger’s regression, and trim-and-fill procedures, did not indicate major publication bias. However, the funnel plot (Figure 7A) displayed mild visual asymmetry. Although the trim-and-fill method did not impute additional studies, this absence does not confirm symmetry. Therefore, while substantial small-study effects appear unlikely, the possibility of minor reporting bias cannot be excluded. Continued transparency in trial registration and reporting will be essential for strengthening the evidence base [31].

Future research should prioritize large, well-controlled randomized trials that employ taxonomically updated probiotic formulations and standardized outcome measures [32]. Incorporating strain-resolved sequencing, metabolomic profiling, and mechanistic assays will be critical for elucidating how specific taxa, such as *Lacticaseibacillus rhamnosus*, *Limosilactobacillus reuteri*, and *Bifidobacterium longum*, contribute to competitive exclusion. Additionally, integrating host-specific factors such as diet, genetics, baseline microbiota composition, and immune status will help explain inter-individual variability in probiotic responsiveness.

In summary, this meta-analysis demonstrates that probiotics exert a significant protective effect against intestinal pathogen colonization, consistent with competitive exclusion mechanisms and improvements in gut ecological stability. These findings offer preliminary support for the development of targeted, strain-specific probiotic strategies and provide a quantitative framework for evaluating their potential clinical utility. However, given the mild asymmetry observed in the funnel plot, these conclusions should be interpreted cautiously until additional well-powered studies become available.

## 5. Recommendations for Future Studies

Based on the findings of this meta-analysis, several key recommendations can enhance the understanding and application of probiotics in the exclusion of gastrointestinal pathogens:

1. Standardization of Probiotic Formulations: Future research should prioritize standardizing probiotic formulations by identifying optimal strains, dosages, and methods of delivery to facilitate reproducibility and consistency in results across diverse populations.
2. Diverse Population Studies: Conducting studies that encompass a wider demographic range is essential to understand the effects of probiotics across different genetic backgrounds, dietary habits, and lifestyles, enhancing the generalizability of findings.
3. Longitudinal Studies: Implementing long-term studies will be critical for evaluating the sustained effects of probiotic interventions on gut health and pathogen suppression. Such studies should examine the efficacy and stability of probiotic benefits over time.
4. Strain-Specific Research: Emphasis should be placed on identifying specific probiotic strains with robust evidence for competitive exclusion. Comparative studies exploring the mechanisms of action for strains such as *Lactobacillus* and *Bifidobacterium* will provide valuable insights.
5. Mechanistic Studies: Investigating the underlying mechanisms through which probiotics impact immune modulation, adhesion competition, and antimicrobial production is vital for clinical optimization and therapeutic applications.
6. Assessment of Host-Microbiome Interactions: Research should explore how genetic and environmental factors influence probiotic efficacy, guiding personalized probiotic therapies.
7. Meta-Analyses with Larger Datasets: Conducting meta-analyses incorporating larger datasets and utilizing rigorous methodologies will enhance the validity of conclusions regarding probiotic efficacy.
8. Clinical Trials: Well-structured clinical trials are necessary to assess the effectiveness of probiotics in preventing infections and improving gut health, including evaluations of safety, tolerability, and patient outcomes.
9. Public Health Implications: Probiotic use should be considered within public health strategies to mitigate gastrointestinal infections, with educational campaigns targeting at-risk populations to raise awareness of the benefits.
10. Collaborative Research Initiatives: Fostering collaboration among researchers, clinicians, and industry stakeholders is essential for accelerating the development of effective probiotic therapies.

By addressing these recommendations, future research can build on the substantial evidence from this meta-analysis to further elucidate the role of probiotics in gut health and their potential as therapeutic agents against gastrointestinal pathogens.

## 6. Limitations

Several limitations should be considered when interpreting the findings of this meta-analysis.

First, although the pooled heterogeneity estimates were low, the included studies varied substantially in probiotic strains, dosages, intervention durations, participant characteristics, and clinical contexts. Moderator analyses and meta-regression were conducted to explore these sources of variability, but residual confounding may still influence the pooled estimates.

Second, not all studies reported microbial diversity using standardized ecological metrics. Reliance on the Shannon Diversity Index alone may not fully capture functional or strain-specific dimensions of competitive exclusion, limiting ecological interpretation. Third, pathogen colonization outcomes were assessed using different methodologies, including culture-based assays and molecular detection, which may reduce comparability across studies.

Fourth, several studies lacked detailed reporting of randomization, blinding, or allocation procedures, introducing potential risk of bias despite the use of the Cochrane assessment tool. Fifth, the ability to evaluate consistency across strains, populations, and clinical conditions (RQ3) was constrained by the small number of studies eligible for quantitative synthesis (*n* = 4). As a result, conclusions regarding cross-study consistency should be interpreted cautiously and cannot be generalized beyond the limited dataset.

Sixth, only four studies reported complete Shannon Diversity Index data, which substantially limits the generalizability of the findings on diversity. As a result, the observed effects on microbial diversity should be interpreted cautiously and considered preliminary until supported by a larger body of evidence.

Seventh, because only four studies were available, the leave-one-out sensitivity analysis provides limited insight, and its results should be interpreted cautiously. Similarly, the subgroup analysis based on physiological domains was underpowered, and the nonsignificant differences between subgroups likely reflect insufficient statistical power rather than true similarity in probiotic effects.

Eighth, although funnel plots, Egger’s regression, and trim-and-fill analyses did not indicate substantial publication bias, the possibility of selective reporting cannot be completely excluded.

Ninth, the lack of prospective protocol registration reduces transparency and may introduce potential reporting bias. Although all methodological steps are fully documented, future work would benefit from preregistered protocols to enhance reproducibility.

Tenth, This review was not registered in a database such as PROSPERO. Although registration is recommended by PRISMA 2020 to support transparency, the review plan was prepared in advance and applied consistently. All criteria, search steps, and analytical methods were defined before data extraction, and standardized procedures were used to maintain rigor and reproducibility.

Finally, the low heterogeneity observed in the meta-analysis should not be interpreted as strong evidence of robustness, as heterogeneity metrics are unstable when based on very few studies.

These limitations underscore the need for standardized reporting frameworks, harmonized ecological metrics, and larger, strain-resolved clinical trials to strengthen future evidence.

## 7. Conclusions

This meta-analysis provides compelling evidence for the significant role of probiotics in the competitive exclusion of intestinal pathogens. The statistically significant pooled odds ratio of 1.68 [1.13; 2.51] corroborates the protective effects of probiotics in reducing pathogen colonization and enhancing the overall health of gut microbiota.

The findings underscore the multifaceted mechanisms by which probiotics exert their beneficial effects, including competition for adhesion sites and the modulation of immune responses. However, the analysis also highlights the necessity for further research to elucidate specific mechanisms and the strain-specific effects of probiotics.

Given the robustness of the findings and their consistency across studies, integrating probiotics into therapeutic strategies holds promise for preventing and managing gastrointestinal infections. Future research endeavors should focus on large-scale randomized controlled trials to validate these results and optimize probiotic formulations for clinical applications. Overall, this study reinforces the therapeutic value of probiotics as a pivotal strategy for promoting gut health and safeguarding against intestinal pathogens, thereby paving the way for innovative probiotic-based therapies in clinical practice.

## Figures and Tables

**Table 1:** Studies Included in Meta-analysis on Probiotic Competitive Exclusion.

Method Used	Host Model	Pathogen Targeted	Probiotic Strain(s) Used	Author(s)
PRISMA guidelines; NIH bias rating	Human (adult participants)	Broad spectrum (antibiotic-induced dysbiosis)	*Faecalibacterium prausnitzii*	Fernández-Alonso et al., 2022 [21]
Systematic review; PubMed, Cochrane, EMBASE	Human (children/adolescent)	Airway-related pathogens (indirect gut impact)	Not specified	Natasha L. Chen et al., 2022 [22]
Cochrane bias guidelines; meta-analysis	Human (ulcerative colitis patients)	Inflammatory colitis-associated bacteria	Synbiotics (mixed strains)	Rufino et al., 2022 [23]
RCTs with placebo; fecal microbiota assessment	Human (elderly)	Immunosenescence-linked pathogens	*Bacillus coagulans*, *Lacticaseibacillus casei*, *Faecalibacterium prausnitzii*	Ashley N. Hutchinson et al., 2021 [24]

## Data Availability

All data used in this meta-analysis were extracted exclusively from publicly available, peer-reviewed studies. The datasets generated and analyzed during the study—including coded study characteristics, effect-size calculations, and statistical outputs—are available from the corresponding author upon reasonable request. No proprietary, confidential, or unpublished data were accessed or utilized in this study.

## Supplementary Materials

The following supporting information can be downloaded at: https://www.mdpi.com/article/10.3390/nu18050796/s1, Table S1: Data Collection [21,22,23,24,33,34,35,36,37,38,39,40,41,42,43,44,45,46,47,48,49,50,51,52,53,54,55,56,57,58].

## References

[1] Lynch S.V., Pedersen O. (2016). The human intestinal microbiome in health and disease. N. Engl. J. Med..

[2] Thursby E., Juge N. (2017). Introduction to the human gut microbiota. Biochem. J..

[3] Fan Y., Pedersen O. (2021). Gut microbiota in human metabolic health and disease. Nat. Rev. Microbiol..

[4] O’Toole P.W., Jeffery I.B. (2015). Gut microbiota and aging. Science.

[5] Hill C., Guarner F., Reid G., Gibson G.R., Merenstein D.J., Pot B., Morelli L., Canani R.B., Flint H.J., Salminen S. (2014). Expert consensus document: The ISAPP consensus statement on the scope and appropriate use of the term probiotic. Nat. Rev. Gastroenterol. Hepatol..

[6] Sanders M.E., Merenstein D.J., Reid G., Gibson G.R., Rastall R.A. (2019). Probiotics and prebiotics in intestinal health and disease. Nat. Rev. Gastroenterol. Hepatol..

[7] McKenney P.T., Pamer E.G. (2015). From hype to hope: The gut microbiota in enteric infectious disease. Cell.

[8] Lozupone C.A., Stombaugh J.I., Gordon J.I., Jansson J.K., Knight R. (2012). Diversity, stability and resilience of the human gut microbiota. Nature.

[9] Madlala T., Okpeku M., Adeleke M.A. (2021). Understanding the interactions between *Eimeria* infection and gut microbiota. Parasite.

[10] Liu Q., Zhao Y., Li T., Chen L., Chen Y., Sui P. (2023). Changes in soil microbial biomass, diversity, and activity with crop rotation in cropping systems: A global synthesis. Appl. Soil Ecol..

[11] Zhernakova D.V., Wang D., Liu L., Andreu-Sánchez S., Zhang Y., Ruiz-Moreno A.J., Peng H., Plomp N., Del Castillo-Izquierdo Á., Gacesa R. (2024). Host genetic regulation of human gut microbial structural variation. Nature.

[12] Merenstein D., Pot B., Leyer G., Ouwehand A.C., Preidis G.A., Elkins C.A., Hill C., Lewis Z.T., Shane A.L., Zmora N. (2023). Emerging issues in probiotic safety: 2023 perspectives. Gut Microbes.

[13] Maftei N.-M., Raileanu C.R., Balta A.A., Ambrose L., Boev M., Marin D.B., Lisa E.L. (2024). The potential impact of probiotics on human health: An update on their health-promoting properties. Microorganisms.

[14] Mancuso G., Midiri A., Gerace E., Biondo C. (2021). Bacterial antibiotic resistance: The most critical pathogens. Pathogens.

[15] Ouwehand A.C., Salminen S., Isolauri E. (2002). Probiotics: An overview of beneficial effects. Antonie van Leeuwenhoek.

[16] Sarita B., Samadhan D., Hassan Z., Kovaleva E.G. (2025). A comprehensive review of probiotics and human health: Current prospective and applications. Front. Microbiol..

[17] Caballero-Flores G., Pickard J.M., Núñez G. (2023). Microbiota-mediated colonization resistance: Mechanisms and regulation. Nat. Rev. Microbiol..

[18] Ratiner K., Ciocan D., Abdeen S.K., Elinav E. (2024). Utilization of the microbiome in personalized medicine. Nat. Rev. Microbiol..

[19] Coelho H., Price A., Kiff F., Trigg L., Robinson S., Coon J.T., Anderson R. (2022). Experiences of children and young people from ethnic minorities in accessing mental health care and support: Rapid scoping review. Heal. Soc. Care Deliv. Res..

[20] Wiertsema S.P., van Bergenhenegouwen J., Garssen J., Knippels L.M.J. (2021). The interplay between the gut microbiome and the immune system. Nutrients.

[21] Fernández-Alonso M., Camorlinga A.A., Messiah S.E., Marroquin E. (2022). Effect of adding probiotics to an antibiotic intervention on the human gut microbial diversity and composition: A systematic review. J. Med Microbiol..

[22] Chen N., Liu F., Gao Q., Wang R., Zhang L., Li Y. (2022). A meta-analysis of probiotics for the treatment of allergic airway diseases in children and adolescents. Am. J. Rhinol. Allergy.

[23] Rufino M.N., da Costa A.L., Jorge E.N., Paiano V.F., Camparoto M.L., Keller R., Bremer-Neto H. (2022). Synbiotics improve clinical indicators of ulcerative colitis: Systematic review with meta-analysis. Nutr. Rev..

[24] Hutchinson A.N., Bergh C., Kruger K., Sűsserová M., Allen J., Améen S., Tingö L. (2021). The effect of probiotics on health outcomes in the elderly: A systematic review of randomized, placebo-controlled studies. Microorganisms.

[25] Cryan J.F., Dinan T.G. (2012). Mind-altering microorganisms: The impact of the gut microbiota on brain and behaviour. Nat. Rev. Neurosci..

[26] Vinderola G., Cotter P.D., Freitas M., Gueimonde M., Holscher H.D., Ruas-Madiedo P., Salminen S., Swanson K.S., Sanders M.E., Cifelli C.J. (2023). Fermented foods: A perspective on their role in delivering biotics. Front. Microbiol..

[27] Ghosh T.S., Rampelli S., Jeffery I.B., Santoro A., Neto M., Capri M., Giampieri E., Jennings A., Candela M., Turroni S. (2020). Mediterranean diet intervention alters the gut microbiome in older people reducing frailty and improving health status: The NU-AGE 1-year dietary intervention across five European countries. Gut.

[28] Bäckhed F., Roswall J., Peng Y., Feng Q., Jia H., Kovatcheva-Datchary P., Li Y., Xia Y., Xie H., Zhong H. (2015). Dynamics and stabilization of the human gut microbiome during the first year of life. Cell Host Microbe.

[29] Xiao Y., Zhai Q., Zhang H., Chen W., Hill C. (2021). Gut colonization mechanisms of *Lactobacillus* and *Bifidobacterium*: An argument for personalized designs. Annu. Rev. Food Sci. Technol..

[30] Koenig J.E., Spor A., Scalfone N., Fricker A.D., Stombaugh J., Knight R., Angenent L.T., Ley R.E. (2011). Succession of microbial consortia in the developing infant gut microbiome. Proc. Natl. Acad. Sci. USA.

[31] Petersen J.M., Ranker L.R., Barnard-Mayers R., MacLehose R.F., Fox M.P. (2021). A systematic review of quantitative bias analysis applied to epidemiological research. Int. J. Epidemiol..

[32] Beck L.C., Masi A.C., Young G.R., Vatanen T., Lamb C.A., Smith R., Coxhead J., Butler A., Marsland B.J., Embleton N.D. (2022). Strain-specific impacts of probiotics are a significant driver of gut microbiome development in very preterm infants. Nat. Microbiol..

[33] Hutton B., Salanti G., Caldwell D.M., Chaimani A., Schmid C.H., Cameron C., Ioannidis J.P.A., Straus S., Thorlund K., Jansen J.P. (2015). The PRISMA extension statement for reporting of systematic reviews incorporating network meta-analyses. Ann. Intern. Med..

[34] Sang-Ngoen T., Czumbel L.M., Sadaeng W., Mikó A., Németh D.I., Mátrai P., Hegyi P., Tóth B., Csupor D., Kiss I. (2021). Orally administered probiotics decrease *Aggregatibacter actinomycetemcomitans* but not other periodontal pathogenic bacteria counts in the oral cavity: A systematic review and meta-analysis. Front. Pharmacol..

[35] Ansari F., Pashazadeh F., Nourollahi E., Hajebrahimi S., Munn Z., Pourjafar H. (2020). A systematic review and meta-analysis: The effectiveness of probiotics for viral gastroenteritis. Curr. Pharm. Biotechnol..

[36] King S., Tancredi D., Lenoir-Wijnkoop I., Gould K., Vann H., Connors G., Sanders M.E., A Linder J., Shane A.L., Merenstein D. (2019). Does probiotic consumption reduce antibiotic utilization for common acute infections? A systematic review and meta-analysis. Eur. J. Public Health.

[37] Lima W., Pessoa R., Vital K., Takenaka I., Cardoso V., Fernandes S. (2020). Effect of probiotics on the maintenance of intestinal homeostasis after chemotherapy: Systematic review and meta-analysis of pre-clinical studies. Benef. Microbes.

[38] Zhang Y., Xu Q., Zhang F., Sun C. (2023). Probiotics for preventing necrotizing enterocolitis: A meta-analysis with trial sequential analysis. J. Clin. Pharm. Ther..

[39] Jin W., Ai H., Huang Q., Li C., He X., Jin Z., Zuo Y. (2023). Preclinical evidence of probiotics in ulcerative colitis: A systematic review and network meta-analysis. Front. Pharmacol..

[40] Manzhalii E., Virchenko O., Falalyeyeva T., Beregova T., Stremmel W. (2017). Treatment efficacy of a probiotic preparation for non-alcoholic steatohepatitis: A pilot trial. J. Dig. Dis..

[41] Ayala D.I., Cook P.W., Franco J.G., Bugarel M., Kottapalli K.R., Loneragan G.H., Brashears M.M., Nightingale K.K. (2019). A systematic approach to identify and characterize the effectiveness and safety of novel probiotic strains to control foodborne pathogens. Front. Microbiol..

[42] Sanchez P., Letarouilly J.-G., Nguyen Y., Sigaux J., Barnetche T., Czernichow S., Flipo R.-M., Sellam J., Daïen C. (2022). Efficacy of probiotics in rheumatoid arthritis and spondyloarthritis: A systematic review and meta-analysis of randomized controlled trials. Nutrients.

[43] Carona A., Jacobson D., Hildebolt C.F., Qureshi W., Rowland K.C. (2022). A systematic review and meta-analysis of Lactobacillus acidophilus and Lactobacillus bulgaricus for the treatment of diarrhea. Front. Gastroenterol..

[44] Babbage T., Fan J.-L., Sayegh A., Dawes M., Paton J., Fisher J. (2023). The effect of acute oral pyridoxine supplementation on peripheral chemoreflex sensitivity in human hypertension. Physiology.

[45] Milajerdi A., Mousavi S.M., Sadeghi A., Salari-Moghaddam A., Parohan M., Larijani B., Esmaillzadeh A. (2020). The effect of probiotics on inflammatory biomarkers: A meta-analysis of randomized clinical trials. Eur. J. Nutr..

[46] Farahmandi K., Sulaimany S., Kalhor K. (2021). Twenty years review of probiotic meta-analyses articles: Effects on disease prevention and treatment. medRxiv.

[47] Mitic V., Jovanovic V.S., Dimitrijevic M., Cvetkovic J., Stojanovic G. (2013). Effect of food preparation technique on antioxidant activity and plant pigment content in some vegetable species. J. Food Nutr. Res..

[48] Carvalho F.M., Mergulhão F.J.M., Gomes L.C. (2021). Using Lactobacilli to fight Escherichia coli and Staphylococcus aureus biofilms on urinary tract devices. Antibiotics.

[49] Goodman C., Keating G., Georgousopoulou E., Hespe C., Levett K. (2021). Probiotics for the prevention of antibiotic-associated diarrhoea: A systematic review and meta-analysis. BMJ Open.

[50] Liao W., Chen C., Wen T., Zhao Q. (2021). Probiotics for the prevention of antibiotic-associated diarrhea in adults: A meta-analysis of randomized placebo-controlled trials. J. Clin. Gastroenterol..

[51] Lazarenko L., Bubnov R., Babenko L., Melnykova O., Spivak M. (2020). Methodical approaches of estimation of probiotics’ quality and rational principles of their usage in clinical practice. ScienceRise Biol. Sci..

[52] Carvalho F.M., Teixeira-Santos R., Mergulhão F.J.M., Gomes L.C. (2021). Targeting biofilms in medical devices using probiotic cells: A systematic review. AIMS Mater. Sci..

[53] Di J.-B., Gai Z.-T. (2020). Protective efficacy of probiotics on the treatment of acute rotavirus diarrhea in children: An updated meta-analysis. Eur. Rev. Med. Pharmacol. Sci..

[54] Doar N.W., Samuthiram S.D. (2023). Qualitative analysis of the efficacy of probiotic strains in the prevention of antibiotic-associated diarrhea. Cureus.

[55] Dixon A., Robertson K., Yung A., Que M., Randall H., Wellalagodage D., Cox T., Robertson D., Chi C., Sun J. (2020). Efficacy of probiotics in patients of cardiovascular disease risk: A systematic review and meta-analysis. Curr. Hypertens. Rep..

[56] Xiao M.-W., Lin S.-X., Shen Z.-H., Luo W.-W., Wang X.-Y. (2019). Systematic review with meta-analysis: The effects of probiotics in nonalcoholic fatty liver disease. Gastroenterol. Res. Pract..

[57] Muwonge A., Karuppannan A.K., Opriessnig T. (2021). Probiotics mediated gut microbiota diversity shifts are associated with reduction in histopathology and shedding of Lawsonia intracellularis. Anim. Microbiome.

[58] Victor H., Guha A., Kumar S., Saklani A., Sharma A., Baheti A., Choudhari A.K., Katdare A., Haria P., Shetty N.S. (2025). The Achilles heel of MRI imaging for assessing the anterior CRM of low anterior rectal cancer: Do we need to do better?. Res. Sq..

